# Coverage of gingival recession using tunnel connective tissue graft technique

**DOI:** 10.4103/0972-124X.55838

**Published:** 2009

**Authors:** Nitin Khuller

**Affiliations:** *Sr. Lecturer, Department of Periodontics, I.T.S Dental College and Hospital, Delhi-Meerut Road, Muradnagar, Ghaziabad - 201 206, UP, India*

**Keywords:** Coronally positioned flap, gingival recession, tunneling procedure

## Abstract

The recession of gingiva is increasingly becoming a more prominent condition in the oral health of many patients and should be treated at its earliest detection. The multi-factorial etiology, decision modality, and current trends followed in treatment of gingival recession are discussed in this presentation. The correction of class I and II gingival recessions are presented as a means of minimizing surgical trauma and achieving predictable aesthetic results. In this case report, I present an alternative technique in treating gingival recession- the tunnel connective tissue graft.

## INTRODUCTION

Gingival recession is a common occurrence and its prevalence increases with age.[1] The recession of gingiva, either localized or generalized, may be associated with one or more surfaces, resulting in attachment loss and root exposure, which can lead to clinical problems such as root surface hypersensitivity, root caries, cervical root abrasions, difficult plaque control, and diminished cosmetic appeal and aesthetic concern. Marginal gingival recession, therefore, can cause major functional and aesthetic problems,[2] and should not be viewed as merely a soft tissue defect, but as the destruction of both the soft and hard tissue. Treatment proposals for this type of defect have evolved based on the knowledge for healing the gingiva and attachment system. The tunnel connective tissue graft is an alternative less invasive quick healing technique for root coverage which has not been reported much in literature.

### Multi-factorial gingival recession etiology

Periodontal marginal tissue recessions have numerous causes, but there is a consensus about the gingival recession etiology.[3]

1. An anatomical condition with a pre-existing or acquired alveolar bone dehiscence combined with localized prominent tooth malposition,[4] inadequate keratinized gingival dimensions in quality and quantity,[5] high muscle attachment, and frenum pull.[6]
2. Occlusal disturbances and para-functional habits. Cervical dental abrasions, also called non-carious cervical lesions (NCCL), have long been thought to be caused by excessive brushing. Controversy over this concept and occlusal etiology still exists. A study was conducted by Miller *et al.,* to verify the occurrence of signs of excessive brushing or occlusal disturbances associated with abfractions.[7] NCCL co-exist almost systematically with occlusal wear facets (94.5%) and lack of canine disclusion (77.2%). The study concluded that clinical signs of excessive brushing were lacking whereas signs of occlusal disturbance were very consistent with the presence of abfractions.
3. Traumatic, overzealous tooth brushing techniques (i.e., forceful, horizontal) frequently associated with a pre-existing lack of cortical bone, or acquired bone dehiscence.
4. Uncontrolled marginal inflammation with accumulation of dental plaque due to improper brushing techniques.
5. Iatrogenic factors related to periodontal, orthodontic[8] and periodontal/restorative procedures on thin biotype (eg, gingivectomy, apically positioned flap, tooth over preparation violating the biologic width, incorrect fitting of the restoration with over-contouring or a gap between the margin of the crown and the tooth structure).[9]
6. No evident clinical etiology in 17% of gingival recessions cases.[10]

### Treatment planning decision modality

If the recession is not progressing and does not provoke tooth sensitivity or poor aesthetics, then tooth-brushing instructions and regular observation through a strict maintenance program would be the optimal treatment. A thorough plaque control is the primary condition for the success of any periodontal surgery. The importance of phase I therapy in the successful treatment of all periodontal surgical procedures includes the ones for root coverage. Phase I therapy for such cases includes both home and in-office care. Maintenance of proper plaque control (both mechanical and chemical) by the patient is of utmost concern to the periodontist. The in-office procedures include thorough scaling and polishing, root planning and a proper periodic recall for assessment of progression of periodontal disease, in this case-gingival recession. Progressive gingival recession in the presence of high thermal sensitivity and/or compromised aesthetic appearance should be treated with surgical root coverage in Class I and II defects.[3] Smoking is a contra-indication for plastic periodontal surgery due to:

- Associated gingival vasoconstriction that often causes necrosis of the soft tissues;
- Lack of adherence of the fibroblasts[11] and
- Alteration in immune response.[12]

The ideal surgical objective is covering the root up to the cemento-enamel junction with a probing depth of less than two mm without probe-induced bleeding. The principal challenge lies in obtaining an excellent blood supply for the covering tissues to avoid possible necrosis and root coverage failure.[13] It is always important to select the periodontal procedure that allows the best aesthetic result, while causing the least amount of trauma.

Miller prescribes complete disclosure at the initial consultation concerning the root coverage that can realistically be obtained through the selected form of treatment[7] [Table 1].

**Table 1:** Miller's classification of recession-type defects[14]

Condition of recession	Success percentage (possible) %
Recession does not extend to the mucogingival junction and is not associated with interdental bone resorption	100
Recession extends beyond the mucogingival junction with no interdental bone resorption	100
Recession is associated with interdental proximal bone resorption and one proximal root exposition	50 to 70
There is mesial and/ or distal proximal bone resorption with exposure of more than one proximal root surface. The papillae are at the same level as the recession	0 to 10

A number of reports published on recession treatment emphasize the size of the pre-surgical defect and its effect on clinical outcomes; in other words, the deeper and narrower the defect, the greater the achieved root coverage. Deeper recessions (i.e., 4 mm or more) had greater attachment level gains than shallow (i.e., less than 4 mm) recessions.[14–17] The mean percentage of root coverage reported for the sub-epithelial CT grafts technique varies between 65 and 98%, while the percentage of complete root coverage ranges from 0 to 90% depending on the recession classification.[18] The position of the interdental papilla should also be taken into consideration as per the classification proposed by Norland and Tarnow.[19]

### Classification for loss of interdental papilla

Nordland and Tarnow[19] proposed a system of classification for the loss of interdental papilla. It utilizes the following identifiable anatomic landmarks:

1. Inter-dental contact point.
2. The facial apical extent of the CEJ.
3. The inter-proximal coronal extent of the CEJ.

Four categories were identified:

*Normal*: Inter-dental papilla fills embrasure space to the apical extent of the inter-dental contact point/area.

*Class I*: The tip of the inter-dental papilla lies between the inter-dental contact point and the most coronal extent of the CEJ. (Space present but CEJ not visible).

*Class II*: The tip of the inter-dental papilla lies at/or the apical to the inter-proximal CEJ but coronal to the apical extent of facial CEJ. (Inter-proximal CEJ visible)

*Class III*: The tip of the inter-dental papilla lies level with or apical to the facial CEJ.

### Various treatment protocols

Numerous procedures and techniques have been designed to provide predictable root coverage in order to solve these problems. Conventional mucogingival surgery includes the following steps:

- The free gingival graft, known to correct mucogingival problems (i.e., lack of keratinized tissues), is used for root coverage.[17,20,21]
- The laterally full pedicle flap will be used if a large and thick strip of keratinized tissue is present on the adjacent teeth.[22,23]
- The split-thickness laterally sliding flap is a modification of the previous procedure.[24]
- The advanced coronally repositioned flap can be used when the keratinized gingival tissue apical to the recession is greater than or equal to 3 mm.

Different modifications have been described including the following

- Two vertical incisions are made extending beyond the mucogingival junction. An extension of the intrasulcular incision, however, can avoid the vertical incision with the interposition of a membrane integrating the guided tissue regeneration (i.e., resorbable and non-resorbable membrane and alloderm) within the root coverage techniques.[16,25]
- The semi-lunar coronally repositioned flap technique requires the oral surgeon to make a semi-lunar incision parallel to the free gingival margin of the facial tissue, a partial dissection, and coronally positioning this tissue over the denuded root.[26]
- The CTG procedure[27–29] permits grafting in a number of ways: Under a flap repositioned to its initial position, under a coronally or a laterally positioned flap, under a double papillae flap, or with a tunnel technique covered by undetached papillae tips.[30]

## CASE REPORT

### Clinical case presentation

Patient selection criteria for the clinical case discussed in this presentation include:

- Non-smoker;
- Patients 18 years and older;
- Buccal recession defects (2.5 mm or greater) classified as either class I or II defects on the maxillary teeth or premolars;
- Radiographic evidence of sufficient interdental bone (i.e., the distance between the crestal bone and the cementoenamel junction is no greater than two mm);
- Clinical indication and/or patient request for recession coverage;
- Gingival thickness of at least 0.5 mm at a point located three mm below the free gingival margin;
- A minimum of two mm of keratinized gingiva; and
- Good oral hygiene [Table 2].

**Table 2:** Prerequisites of gingival recession surgery

Indications	Contraindications
Small amount of keratinized gingiva	Insufficient or inefficient
Class I or II recessions	Smoking patients
Aesthetic concerns	Desquamative gingivitis
Single or multiple recessions	
Root hypersensitivity

The selected teeth were vital, free of restorations, bleed-free upon probing after the initial preparation, and had not been treated surgically for at least two years. After obtaining adequate anesthesia, the exposed root surfaces of patients were scaled and planed utilizing ultrasonic hand instruments. The root surfaces were then reshaped with a smooth diamond bur and polished.

### Tunnel connective tissue graft (TCTG)[31,32]

**Figure 1:**
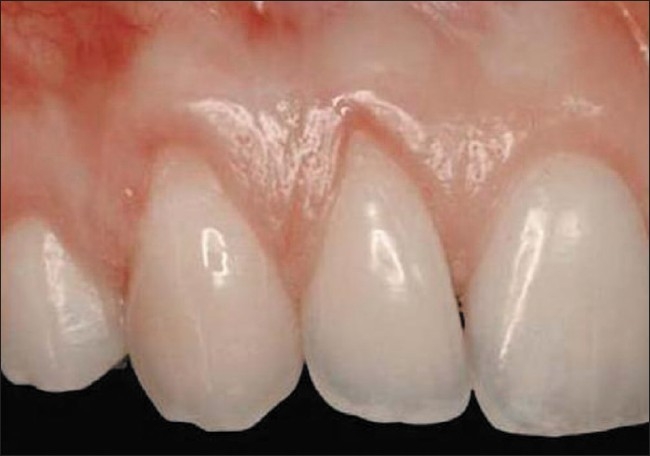
Class I gingival recession on the right maxillary canine and lateral incisor

**Figure 2:**
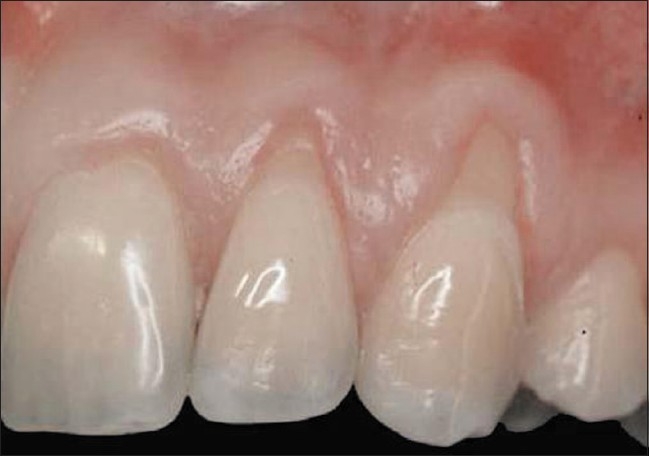
Class II gingival recession on the left maxillary canine and lateral incisor

**Figure 3:**
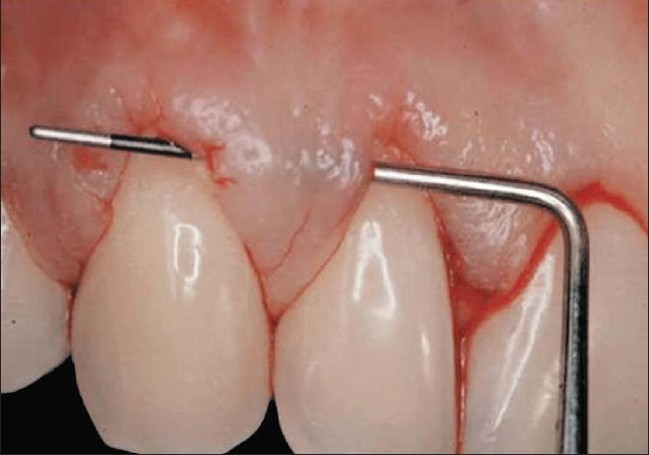
Transversal sounding of the tunnel without detaching the peak of the papillae

**Figure 4:**
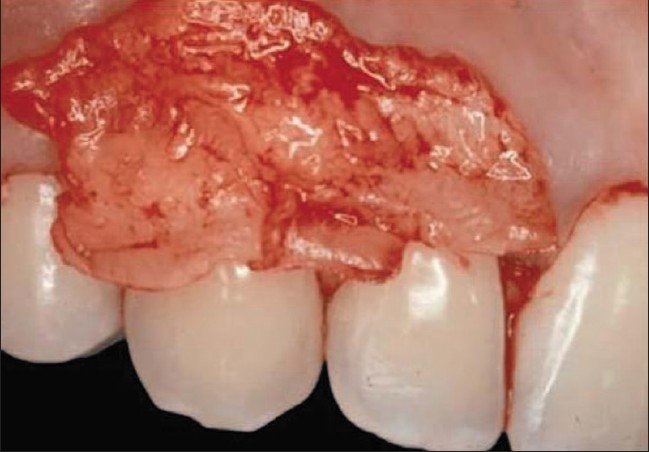
Large and thick CTG after palatal harvesting

**Figure 5:**
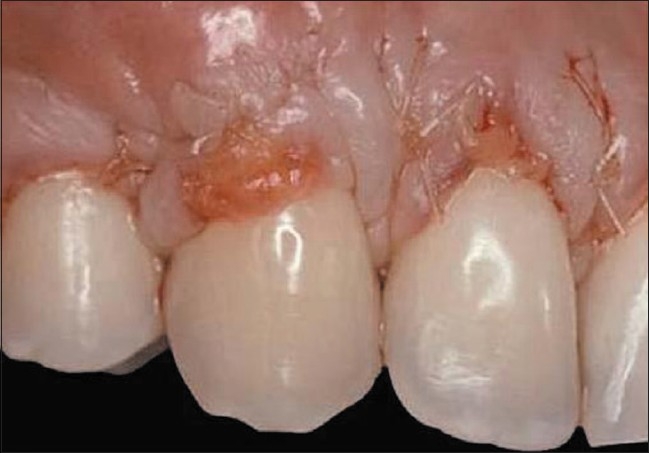
The CTG is inserted in the tunnel and the flap is advanced and sutured with the graft

**Figure 6:**
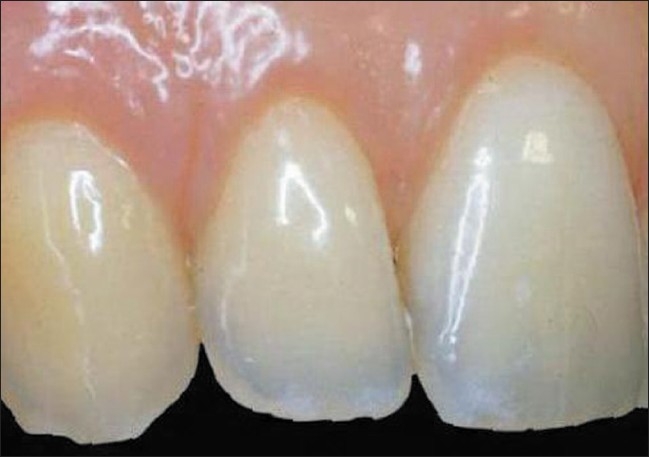
Right maxillary six months post-operative

A 19-year-old female presented whose chief complaint was root sensitivity and poor aesthetics on her maxillary lateral incisors and canines [Figures 1 and 2]. The tunnel technique was selected to treat both sides simultaneously presenting with class I and II gingival recession [Figures 3 and 4]. A sulcular incision was designed on both sides, from the first premolar to the central incisors, and a partial dissection was carefully performed in order to create a deep pouch beyond the mucogingival junction while keeping the tip of the interproximal papillae attached to the teeth below the proximal contact point. A primary flap on the right and left palatal sites with one line of incision allows the harvesting of thick, sizable connective tissue [Figure 5]. The primary flap was immediately sutured to prevent bleeding [Figure 6]. The CTG, using 4-0 sutures, was delicately inserted inside the pouch and was then stabilized with the flap using 5-0 Vicryl sutures. A periodontal pack was placed and the patient was advised not to brush for 72 hrs in the area of surgery. An ice-pack was given immediately post-operatively and asked to restrain from spitting or rinsing for first few hours. The patient was recalled after 24 hrs, three days and then after one week. The pack was removed, the area of operation appeared normal without any trace of sloughing. The patient was asked to report to the clinic every month for six months for routine check up. The healing progressed uneventfully and the gingival recession was totally covered with a beautiful aesthetic result on both sides [Table 3].

**Table 3:** Tunnel connective tissue graft

Advantage	Disadvantage
Excellent adaptation on the recipient site	Traumatic surgery for the patient
Highly aesthetic results	Requires two surgical sites
High vascularization by the advanced flap	Delicate harvesting of the grafts
Increase thickness of the keratinized gingiva	Difficult stabilization of the graft
Harmony in the gingival colour/texture	Palatal graft has limited quantity and thickness
	Lengthy surgery/healings

## DISCUSSION

There are four basic techniques for root coverage: (1) pedicle grafts, (2) free gingival grafts, (3) connective tissue grafts, and (4) membrane barrier guided tissue regeneration technique. All of these methods are used frequently today, and the use of each one of them is based on its advantage or disadvantage, as well as on the individual surgeon's preference and experience.

The basic rationale of the pedicle graft technique is to cover the exposed avascular root surface with a contiguous (in contrast to free) soft tissue auto-graft from an adjacent site. The best-known technique is the “laterally positioned pedicle graft”, which was introduced by Grupe and Warren in 1956;[25] this represents one of the first in the series of procedures of mucogingival surgery designed to cover exposed root surfaces.

The free gingival graft procedure involves a keratinized epithelial graft obtained from the palate or an edentulous ridge and its placement in the recession area. Hattler was the first to utilize the keratinized gingiva of the interdental papillae to cover denuded root surfaces.[33] The technique was popularized by Sullivan and Atkins, who described the specifics and principles of the free gingival graft technique, as well as its biologic aspects of wound healing.[17]

The free connective tissue graft is a bilaminar procedure designed to maximize the supraperiosteal and gingival blood supply to the grafted tissue. The graft is placed over the recession area, while nutrients and revascularization are derived from the recipient bed, interdental papillae, and the overlying flap. The use of free connective tissue for root coverage was introduced by Edel in 1974, but it did not receive wide approval by the profession.[34] Later, the technique was presented by Langer and Calagna as the “subepithelial connective tissue graft” and described in detail by Langer and Langer.[30,35,36]

In cases of deep recessions, the flap may be coronally positioned to provide greater coverage and better blood supply to the connective tissue graft.[37,38] Another version of the connective tissue graft is the “subpedicle connective tissue graft”, presented by Nelson and further modified by Harris.[39,40] The rationale behind this approach is to provide optimal nutrients to the connective tissue lining of the root surface. The “envelope technique” is another version of the connective tissue graft.[41] The graft is placed directly on the denuded root surface, while its major part is inserted into a recipient bed prepared by split-thickness dissection without a flap elevation. This technique is indicated only in single-tooth recessions.

The surgical technique of choice depends on several factors, each having advantages and disadvantages. The clinician should choose from among the different surgical protocols available, selecting the least traumatic to the patient. I found this technique, i.e. the tunnel connective tissue graft technique to be a viable option for root coverage in Miller's class I and II type of gingival recession. However, more studies using greater number of patients should be done to determine its advantages and disadvantages in the long run.
